# The effect of reducing work rate on total work done and time to task failure during severe intensity exercise

**DOI:** 10.1007/s00421-025-06046-z

**Published:** 2025-11-12

**Authors:** Alexander J. Welburn, Stephen J. Bailey, Richard A. Ferguson

**Affiliations:** School of Sport, Exercise and Health Sciences, Loughborough University, Loughborough, LE11 3TU UK

**Keywords:** Intermittent exercise, Cycling performance, Power-duration relationship, *W′* balance, Pacing

## Abstract

**Purpose:**

This study assesses the finite work capacity (*W′*) above critical power (CP) when work rate is reduced during severe intensity exercise and whether reducing work rate immediately prior to task failure allows continued work above CP.

**Methods:**

14 cyclists performed three experimental trials. Two consisted of exercising at a fixed work rate (WR) that depleted 70% of *W′* in 90 s and 180 s respectively, after which work rate was reduced to CP + 20 W and continued until task failure. The third consisted of exercising at a fixed work rate to achieve task failure in 5 min (P_5_) when work rate was reduced, and exercise continued until task failure. Work rate was reduced a further two times and continued until task failure. Predicted (WORK_PRED_) and actual work above CP (WORK_90_, WORK_180_, WORK_P5_) and predicted (TTE_PRED_) and actual time to task failure (TTE_90_, TTE_180_, TTE_P5_) for each of the trials were compared.

**Results:**

Compared to WORK_PRED_, total work above CP was greater (*P* < 0.05) in all three trials [WORK_90_ (+ 5.4 ± 0.7 kJ); WORK_180_ (+ 4.3 ± 1.3 kJ); WORK_P5_ (+ 2.7 ± 0.4 kJ)]. Compared to the respective predicted time, time to task failure was greater (*P* < 0.05) in all three trials [TTE_90_ (+ 256 ± 38 s); TTE_180_ (+ 240 ± 55s); TTE_P5_ (+ 109 ± 12s)].

**Conclusion:**

*W′* is not a fixed parameter. When *W′* was partially depleted work above CP continued more than predicted. Moreover, when *W′* is theoretically 0 kJ, work can continue above CP, if work rate is reduced.

## Introduction

The hyperbolic relationship between power output and time to exhaustion during high-intensity exercise, can be described by a power asymptote, critical power (CP), and the curvature constant *W′*. CP demarcates the heavy and severe intensity domains, reflecting the highest sustainable rate of oxidative metabolism (Jones et al. 2010). *W′* is considered a fixed and finite amount of work that can be completed above CP. Both parameters allow for prediction of task failure during constant load exercise in the serve intensity domain(Fukuba et al. 2003; Morton 2006; Poole et al. 1988) and are key cycling performance parameters (Chorley and Lamb 2020). Knowledge of CP and the behaviour of *W′* can help ensure that *W′* is fully depleted where required and not prematurely e.g. in the team pursuit (Pugh et al. 2022; Schumacher and Mueller 2002), forming a break away(Abbiss et al. 2013) or pacing a time trial (Smith et al. 1999).

The classic interpretation is that *W′* represents a fixed anaerobic work capacity (Moritani et al. 1981). However, this observation has been challenged, and *W′* is now thought to be a ‘fatigue cascade’ of metabolic processes during supra-CP exercise (Murgatroyd et al. 2011). One of the underlying assumptions with the CP/*W′* model is that when *W′* has been fully depleted exercise is terminated due to task failure, or work rate must drop below CP (Hill 1993; Jones and Vanhatalo 2017; Morton 2006; Poole et al. 1988). However, a series of studies have challenged this notion, in which changes in work rate have altered the limits of exercise tolerance i.e. the point in which exercise would be terminated exceeded the duration originally predicted from the CP/*W’* model.

Initially, Fukuba et al. (2003) observed that the total amount of work performed above CP (i.e., *W′*) was not affected by work rate variations (after 50% of *W′* had been depleted) during exhaustive cycling exercise in the range of 117% and 134% of CP. In contrast, Dekerle et al. (2015) demonstrated that after *W′* was depleted by 70% during 3 minutes of sustained exercise at ~ 140% of CP, 20% more work was performed than the CP model predicted when the work rate was reduced to just above CP. However, this increase in work done was not observed when the same amount of *W’* was depleted over a 10-minute duration (i.e., at ~ 112% of CP. These data suggest that the initial rate of *W′* depletion may be a determining factor for the increase in work capacity above CP and exercise tolerance. Studies have also been conducted in which exercise is taken to task failure at which point the work rate is reduced. Initially, Coats et al. (2003) suggested that following exercise at a work rate designed to attain exhaustion in 6 min, an abrupt reduction in work rate at the point of task failure exercise could not be maintained if the work rate was above CP (110% of CP) but was able to continue if the work rate was reduced to below CP (90% of CP). Despite these conclusions, when work rate was dropped to 110% of CP participants were able to continue for a further ~ 30 s. Subsequently, Chidnok et al. (2013) demonstrated that following 3 min of single leg exercise performed to the point of task failure (at ~ 153% of CP), exercise was able to continue for ~ 39 s even when the work rate was reduced but still above CP (~ 123% of CP).

Taken together, whilst the evidence is contradictory, these observations suggest that *W′* may not be fixed and work can continue when the work rate is reduced but still exceeds CP. Insight into the duration in which the limits of exercise tolerance can be exceed with a reduction in work rate are not fully known. Further understanding the fixed nature of *W′* and the boundary of its predictive ability could provide greater insights and understanding of *W′*. This will have implications for predicting exercise tolerance during variable efforts and the modelling of *W′* depletion/reconstitution (*W′*_BAL_).

Therefore, the aim of this study was two-fold: 1) assess the total work done above CP when the work rate is reduced during severe intensity exercise; (2) assess if reducing the work rate immediately prior to task failure allows work to continue above CP. It was hypothesised that: (1) there would be an increase in work done vs. predicted when work rate was reduced following 70% of *W′* depletion; (2) work rate would be sustainable past the predicted time to exhaustion, despite work rate being reduced.

## Methods

### Participants

Fourteen healthy participants (9 males, 5 females, age: 21 [2] y, height, 1.80 [0.10] m; body mass, 67.8 [7.4] kg; mean [SD]; Table 1) volunteered to participate in the laboratory-based investigation. Participants were competitive cyclists training between 6 and 15 h per week, competing at regional and national level. All completed health screening questionnaires before participation to mitigate for contraindications to maximal exercise. Participants did not have a history of cardiovascular, haematological, neuromuscular, or musculoskeletal abnormalities. Participants were fully informed of the risks and discomforts associated with all experimental trials before providing written, informed consent. All experimental procedures were approved by the Loughborough University Ethics Approvals Human Participants Sub-Committee (2022-11036-5519), and conformed to the Declaration of Helsinki, except for registration in a database.

### Experimental protocol

Participants attended the laboratory on seven separate occasions for the determination of V̇O_2max_, MAP, CP, *W′* and three severe domain exercise trials involving stepwise reductions in work rate to task failure. All exercise tests were performed on an electronically braked ergometer (Lode Excalibur Sport) setup to match the participant’s own measurements then replicated across all visits. Exercise tests were conducted at the participants freely chosen pedal cadence and continued until task failure or when cadence fell 10% below the chosen cadence for ~ 5 s, despite strong verbal encouragement. Prior to each performance trial (except V̇O_2max_) participants performed a standardised warm-up protocol involving 5-min at 50 W and 100 W, before completing 3 min at 55% and 65% of MAP, before a final 5 min at 50 W. Participants were instructed to maintain a normal diet during the testing period and refrain from consuming alcohol and caffeine during the 24 h preceding testing. All tests were conducted in constant laboratory ambient conditions (19–21 °C, 40–50% humidity).

### Test procedures

#### V̇O_2max_ and MAP

Participants completed an incremental step test to determine V̇O_2max_ and MAP. Following a warm-up for 5 min at 50 W, the test began at 150 W for males 100 W for females for 5 min after which power increased 5 W every 12 s for a smooth linear work rate increase, which equated to 25 W every 60 s until task failure. Breath-by-breath pulmonary gas exchange was measured continuously throughout the exercise (Vyntus-CPX; CareFusion, Hoechberg, Germany). The system had been calibrated with known O_2_ and CO_2_ concentrations and a 3-L volume syringe. V̇O_2max_ and MAP were defined as the highest V̇O_2_ for 30 s and power output achieved for 60 s period during the test, respectively.

#### CP and W′

Participants performed a minimum of 3 constant-load tests that were continued until the limit of tolerance at between 80 and 105% of MAP, the sequence of which was randomised. These were designed to elicit exhaustion within 2- to 15-min (Jones et al. 2019). Time to task failure (t) was recorded to the nearest second. No feedback regarding the power output or times achieved were provided, however participants were permitted to view pedal cadence throughout. The parameters of the power-duration relationship (CP and *W′*) were calculated using the inverse linear model (Eq. 1), the linear work-time model (Eq. 2) and the hyperbolic model (Eq. 3). The equation associated with the lowest combined standard error for each participant was selected.

1 $$\:\mathrm{P}=\left(\frac{\mathrm{W}^{\prime\:}}{{1/\mathrm{t}}_{\mathrm{l}\mathrm{i}\mathrm{m}}}\right)+\mathrm{C}\mathrm{P}$$

2 $${\mathrm{W}} = \left( {{\mathrm{CP}} \cdot {\mathrm{T}}} \right) + W^\prime$$

3 $$\:T=\frac{{W}^{{\prime\:}}}{(\mathrm{P}-\mathrm{C}\mathrm{P})}$$

where P is the given power output above CP, t is the time to task failure (s), *W′* is the total work (J) completed above CP, W is the total work done (J), CP is critical power (W).

### Experimental *W′* depletion trials

**Fig. 1:**
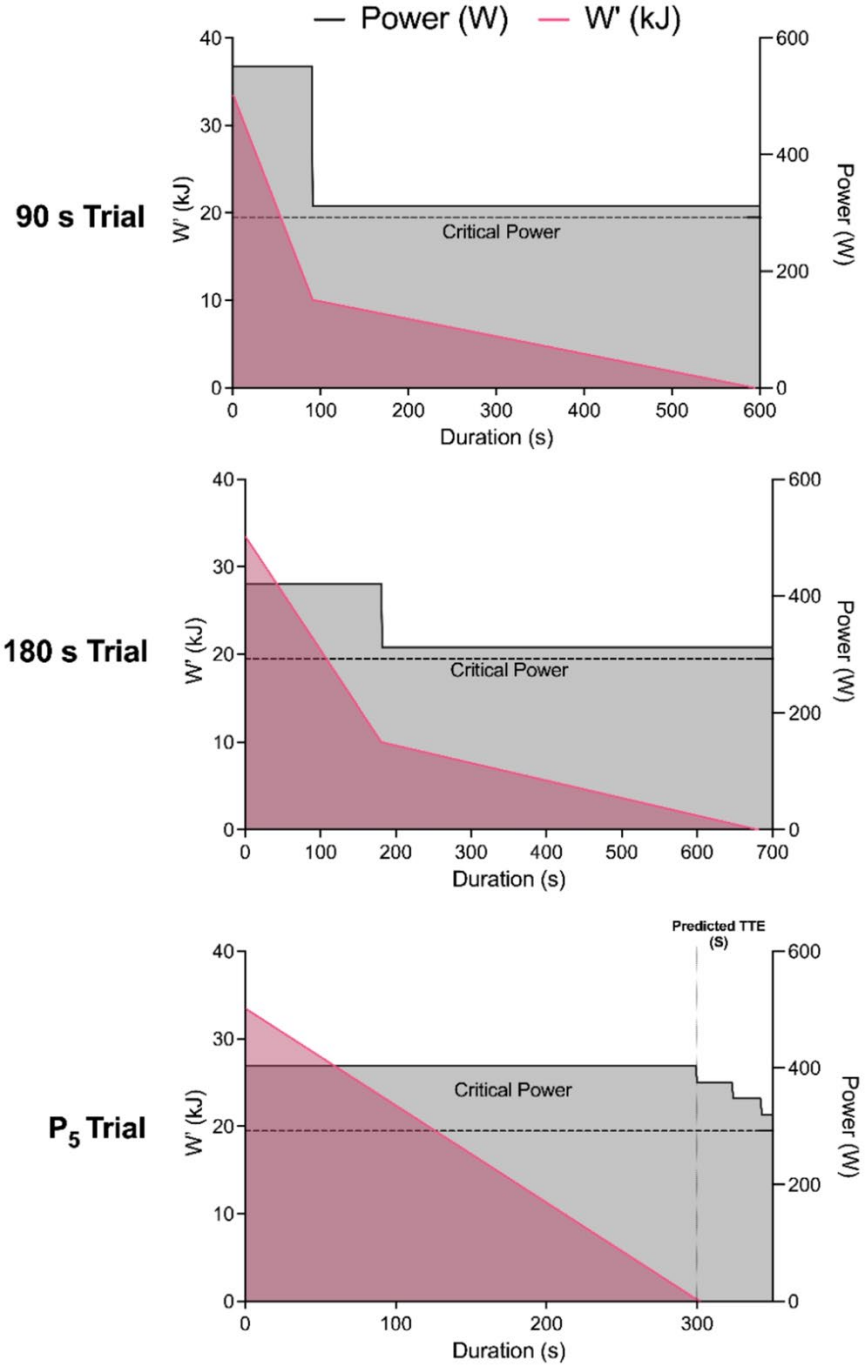
Experimental schematic of work rate and *W′* depletion for the three experimental trials

The first two trials (Fig. 1) consisted of exercising at a fixed work rate, resulting in the depletion of 70% of *W′* in 90 s and 180 s, respectively. The work rate for each trial was calculated as shown in Eq. 4.

4 $${\text{Work rate}}=\left( {{{\left( {{W^\prime }*0.{\mathrm{7}}} \right)} \mathord{\left/ {\vphantom {{\left( {{W^\prime }*0.{\mathrm{7}}} \right)} {{\text{duration }}\left( {\mathrm{s}} \right)}}} \right. \kern-0pt} {{\text{duration }}\left( {\mathrm{s}} \right)}}} \right)+{\mathrm{CP}}$$

After the 70% depletion was completed, the work rate was reduced to CP + 20 W and exercise continued until task failure. This was based the protocol described by Dekerle et al. (2015), who used CP + 10 W following partial *W′* depletion. To ensure that exercise remained within the severe-intensity domain, even when accounting for the potential measurement error in determining CP, we selected CP + 20 W as the lowest work rate expected to reliably achieve this. The third trial (Fig. 1) consisted of exercising at a fixed work rate to achieve task failure in 5 min (P_5_). At this point, when pedal cadence began to reduce (by approximately ~ 5 rpm, a typical indication of the onset of task failure) the work rate was rapidly reduced by a work rate equal to 25% of the difference between CP and P_5_ and exercise continued until pedal cadence began to reduce again. This reduction in work rate (also 25% of the difference between CP and P_5_) was repeated a further two times (three work rate reductions in total) after which exercise continued until task failure. To reduce potential bias within participants, the researcher was blind to exercise duration in the final 60 s of the trial and only able to view pedal cadence.

Time to task failure was predicted (TTE_PRED_) which, based on the CP/*W’* model, was different for each trial. Actual TTE was measured for each trial (TTE_90_, TTE_180_, TTE_P5_). Total work above CP was predicted based on the CP/*W’* model (WORK_PRED_) which was the same for each trial (i.e., *W’*). Total work above CP was measured during each trial (WORK_90_, WORK_180_, WORK_P5_).

### Statistical analysis

A one-way ANOVA was performed to compare WORK_PRED_, WORK_90_ and WORK_180_. WORK_P5_ was not included in this analysis due to methodological differences in *W′* depletion (partial vs. full depletion) but was compared with WORK_PRED_ using a paired *t-test*. A one-way ANOVA was performed to compare TTE_PRED_ in the respective trials and TTE_90_, TTE_180_, TTE_P5_. Where significant effects were observed, Bonferroni corrected post-hoc *t-tests* were used to locate differences. When Mauchly’s test of sphericity indicated that the assumptions of sphericity had been violated, Greenhouse-Geisser correction was used. Statistical significance was accepted at *p* < 0.05 and data are presented as mean [SD].

## Results

Participant performance parameters and the associated CP/*W′* equation used to provide the lowest combined standard error are shown in Table 1. A summary of work rate and step reduction parameters for the three experimental *W′* depletion trials are shown in Table 2.

**Table 1:** Performance characteristics of the P-T_lim_ relationship and performance values

ID	Sex	CP/W′ equation	CP (W)	CP SE (W)	W′ (kJ)	W′ SE (kJ)	MAP (W)	V̇O_2max_ (mL·min^−1^·kg^−1^)
1	M	3	292	7	33.4	5.1	422	52.2
2	M	3	296	1	22.4	1.1	400	64.5
3	F	3	189	4	11.4	2.3	260	47.2
4	M	3	307	2	33.5	1.3	439	59.3
5	M	3	305	4	31.3	6.6	459	68.3
6	M	3	296	0	20.3	0.3	403	74.3
7	F	3	176	1	16.3	0.6	268	44.4
8	F	3	178	2	13.7	0.8	257	43.2
9	M	3	243	2	19.6	1.3	338	64.7
10	M	3	299	3	21.6	1.8	419	66.8
11	M	3	207	2	20.2	0.8	310	42.0
12	F	3	231	4	19.4	1.5	342	53.0
13	M	3	319	1	31.3	0.4	451	53.1
14	F	1	203	4	13.0	1.0	290	56.7
Mean			253	3	21.9	1.8	364	56.7
SD			54	2	7.6	1.9	72	10.2

CP, critical power MAP, maximal aerobic power; SE, standard error; V̇O_2max,_ maximal oxygen uptake; *W′*, curvature constant

**Table 2:** Summary of depletion trial parameters

	90 s trial	180 s trial	*P*_5_ trial
Work rate (W)	424 ± 108	345 ± 80	330 ± 74
Work rate % of CP	166 ± 13	135 ± 27	129 ± 7
CP + 20 W (W)	273 ± 54	273 ± 54	-
Work rate reduced at (s)	-	-	302 ± 10
Work rate reduction per step (W)	-	-	18 ± 6

**Fig. 2:**
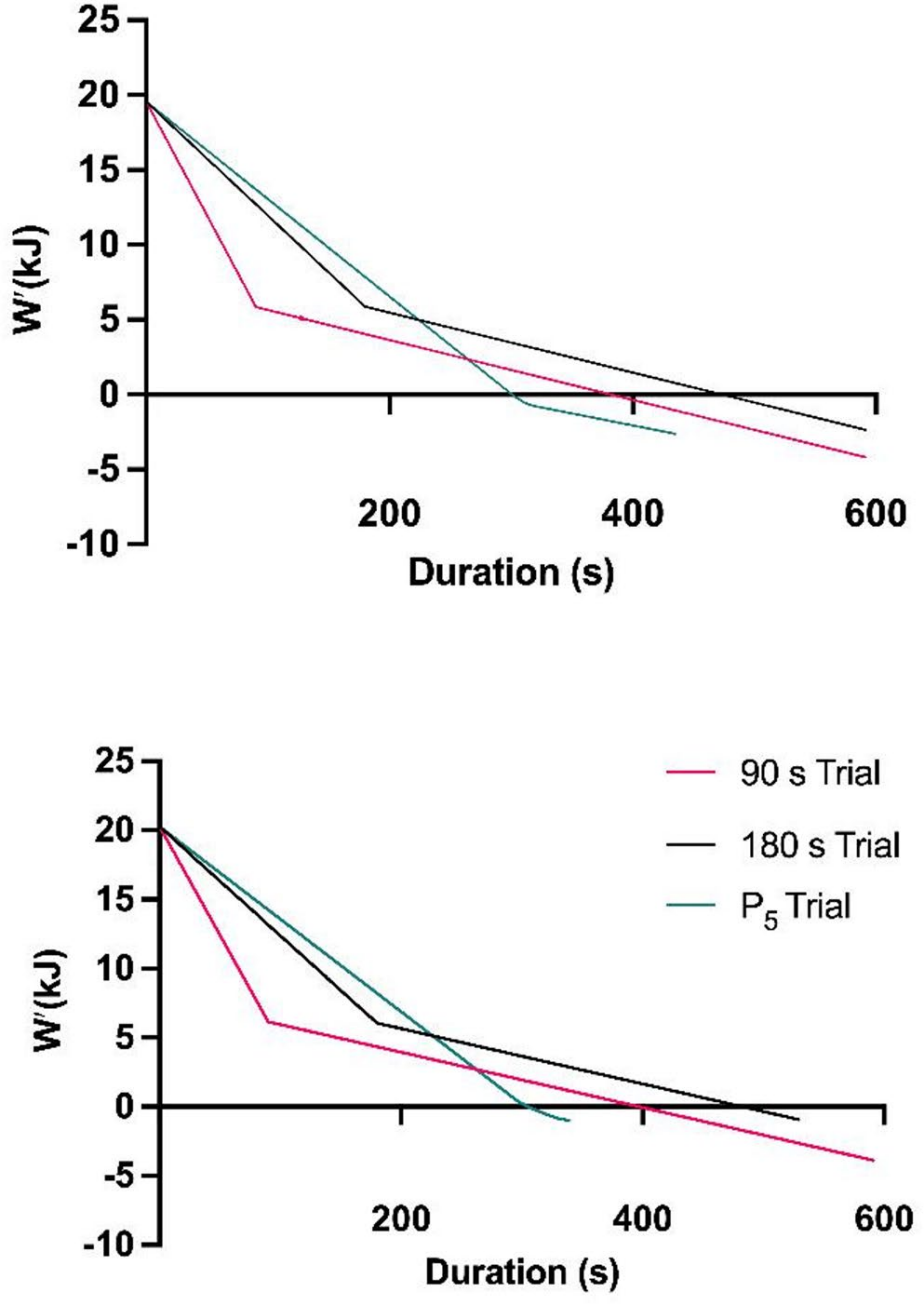
*W’* depletion in two participants with different CP characteristics throughout the three experimental trials. Top (male participant 9), CP = 243 W, *W′* 19.6 kJ; Bottom (male participant 6], CP = 296 W, *W′* 20.3 kJ

Visual representations of *W′* depletion for the three trials in two different participants are shown in Fig. 2.

**Fig. 3:**
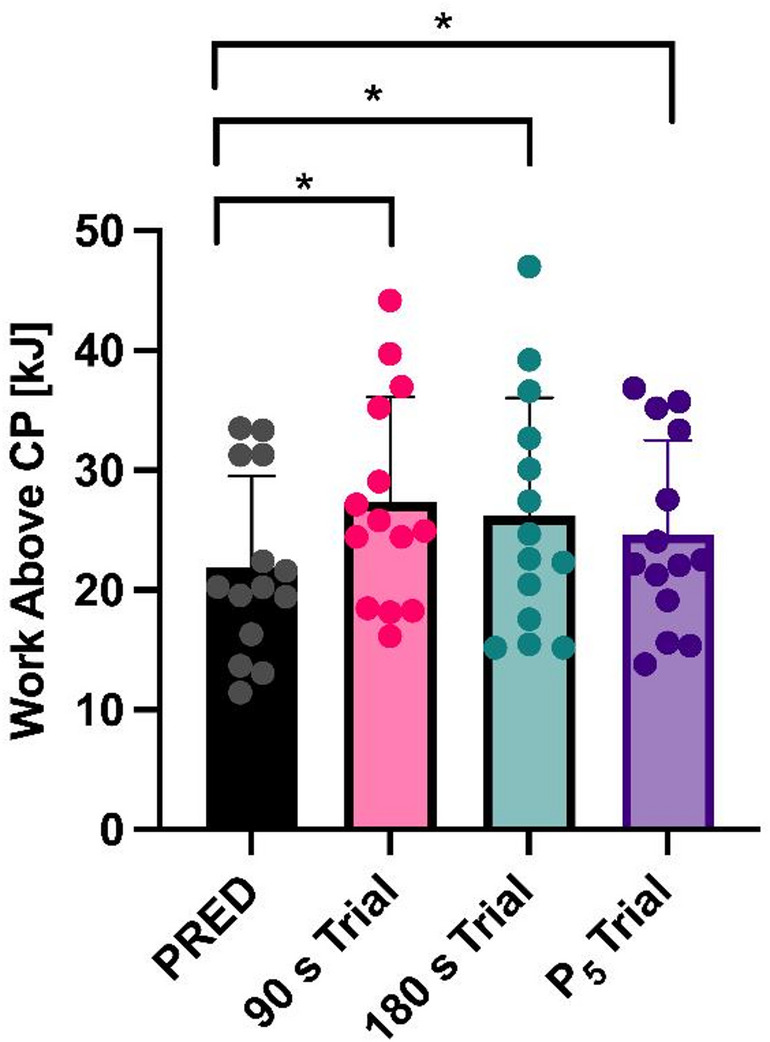
Total work above CP performed in the three trials compared to predicted. Mean ± SD and individual data are shown. * *P* ≤ 0.05

Total work above CP in the three trials was greater (F_(1.264,16.4)_ = 8.144, *P* = 0.008) than predicted (Fig. 3). Specifically, compared to WORK_PRED_, total work above CP was greater in all three trials [WORK_90_ (+ 5.4 ± 0.7 kJ, *P* < 0.001); WORK_180_ (+ 4.3 ± 1.3 kJ, *P* = 0.032); WORK_P5_ (+ 2.7 ± 0.4 kJ, *P* < 0.001)]. There was no difference (*P* = 0.951) between WORK_90_ and WORK_180_.

**Fig. 4:**
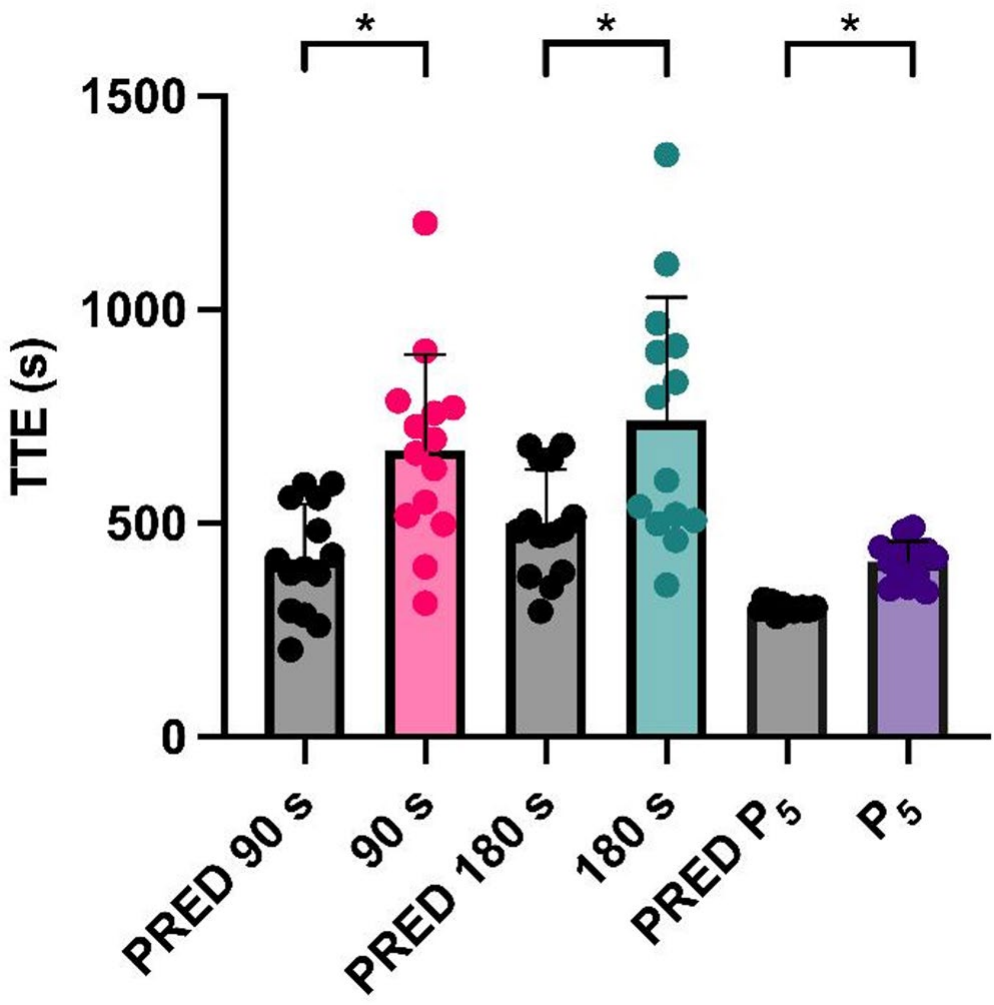
Time to task failure in the three trials compared to predicted. Mean ± SD and individual data are shown. * *P* ≤ 0.05

Time to task failure in all three trials was greater (F_(2.145,27.89)_ = 18.86, *P* < 0.001) than predicted. (Fig. 4). Specifically, compared to the respective predicted time for each of the three trials (90 s, 180 s and P_5_) time to task failure was greater in all three trials [TTE_90_ (+ 256 ± 38 s, *P* < 0.001); TTE_180_ (+ 240 ± 55s, *P* = 0.003); TTE_P5_ (+ 109 ± 12s, *P* < 0.001)]. There was no difference (*P* = 0.891) between TTE_90_ and TTE_180_.

## Discussion

The main findings of this study are as follows: (1) *W′* is not a fixed parameter and fails to predict exercise tolerance when work rate was reduced but still above CP following 70% of *W′* depletion; (2) there was no difference in total work done above CP at task failure between the 90–180 s *W′* depletion trials; (3) work rate was sustainable past the predicted time to exhaustion (and *W′* was theoretically 0 kJ), despite work rate being reduced at the onset of task failure.

One of the underlying assumptions is that *W′* is fixed irrespective of the rate in which it is being utilised (Morton 2006; Skiba et al. 2012) such that when *W′* is 0 kJ task failure will occur, or work rate must drop below CP. The present findings confirm previous observations and collectively demonstrate that *W′* is not a fixed parameter, whereby when *W′* was partially depleted work above CP continued more than predicted by the CP/*W′* model after the initial work rate was reduced. Moreover, the work rate at which *W′* was depleted (equivalent to 166 and 135% CP) did not influence the additonal work done. These are similar to the observations made by Dekerle et al. (2015) whereby the depletion 70% of *W′* over a 3 min duration (at a work rate equivalent of 140% CP) resulted in more work above CP being performed wen the work rate was reduced. It is of interest to note that Derkele also demonstrated that when the work rate at which *W′* was depleted was lower (equivalent to 112% of CP) and over a longer duration (10 min) there wasno difference in total work above CP compared to predicted. Similarly, exercising as a lower work rate (117–134%) of CP, and depleting 50% of *W′* also did not alter total work above CP (Fukuba et al. 2003). It is therefore possible that during variable intensity exercise (above CP) *W′* may, in fact, be dependent on work rate and not independent of it, as proposed (Morton 2006).

Additional support for the hypothesis that *W′* is not fixed is provided from the observation in the present study that work above CP was able to continue at the point of task failure, where *W′* is theoretically fully depleted. This supports the work of Coats et al. (2003), who despite reporting that after reaching the point of task failure (and theoretically depleting *W′*) the work rate needed to be reduced below CP for exercise to continue yet demonstrated that a further ~ 30 s (12–43 s) of work above CP was possible. This clearly challenges the assumption that when ‘exhaustion’ occurs, work rate must drop below CP (Morton 2006; Poole et al. 1988). This may be partially explained if the 3-parameter model is considered (Morton 1996) which indicates that the effective work capacity above CP (i.e., the usable portion of *W′*) decreases as power output approaches CP (Vinetti et al. 2019). However, this property alone cannot account for the observed increase in *W′*.

The mechanisms that explain the increase in exercise tolerance following the initial *W′* depleting periods of exercise are not fully understood but maybe due to factors similar to the ‘priming’ effect of prior intense exercise which results in an increase in the contribution of aerobic and reduced reliance on anaerobic energy production (Bangsbo et al. 2001; Burnley et al. 2005) and a consequent reduction in the associated fatigue metabolite accumulation/depletion. Furthermore, there may be an excess muscle perfusion during the transition between the two work rates (e.g., Towse et al. 2005) which could also facilitate a transient increase in the removal of fatigue-related metabolites and/or partial PCr resynthesis. Collectively these findings have implications for pacing strategies during severe intensity efforts when a variable work rate is necessary yet still required to be above CP (e.g., team pursuit cycling; Pugh et al. 2022) and whether a ‘fast start’ or ‘all out’ pacing strategy is optimal (Aisbett et al. 2003, 2009; Brock et al. 2018). Critically, these observations also have implications for the accuracy of modelling of *W′* depletion and recovery, using the *W′*_BAL_ model (Skiba et al. 2012), which is assumes *W′* is a fixed parameter.

This study is not without limitations. Inclusion of a standalone validation exercise bout performed at CP + 20 W without any preceding depletion effort would have confirmed that the additional total work done above CP past that predicted was in-fact due to the initial depletion. Furthermore, small errors in the calculation of CP and *W′* may influence both the predicted and actual work done during each exercise trial. However, we were careful to use the CP/*W′* equation associated with the lowest combined standard error (Hill and Smith 1994). We did not assess pulmonary gas exchange or other physiological measurements which limits the mechanistic understanding of our observations.

In conclusion, these data support the proposition that *W′* is not a fixed parameter, under circumstances of partial and complete *W′* depletion. When *W′* was partially depleted work above CP continued more than predicted after the initial work rate was reduced. It is also demonstrated that when *W′* is theoretically 0 kJ (i.e., task failure has been reached) work can continue above CP, with reductions in work rate. We hereby define this ability to maintain a work rate above CP more than predicted by the CP/*W′* model as a *residual capacity of W′* (*W′*_RES_).

## Data Availability

Data are available upon reasonable request. Declarations.

## Abbreviations

CP: Critical power
MAP: Maximal aerobic power
P_5_: Power output to attained exhaustion in 5 min
PCr: Phosphocreatine
TTE: Time to exhaustion
TTE_180_: Time to exhaustion in the 180 s trial
TTE_90_: Time to exhaustion in the 90 s trial
TTE_P5_: Time to exhaustion in the P_5_ trial
V̇O₂_max_: Maximal oxygen uptake
Wʹ: Curvature constant
Wʹ_BAL_: Balance between Wʹ depletion and Wʹ reconstitution during intermittent exercise
W′_RES_: Wʹ residual capacity
WORK_180_: Actual work above CP completed in 180 s trial
WORK_90_: Actual work above CP completed in 90 s trial
WORK_P5_: Actual work above CP completed in P_5_ trial
WORK_PRED_: Predicted amount of work above CP
